# Data from the Swiss National Arthroplasty Registry SIRIS suggest that unicompartmental knee arthroplasty is associated with a lower risk of periprosthetic joint infection than total knee arthroplasty

**DOI:** 10.1007/s00402-025-06156-5

**Published:** 2026-03-04

**Authors:** Peter Wahl, Christian Brand, Bernhard Christen

**Affiliations:** 1 Department of Biomedical Engineering, University of Basel, Allschwil, Switzerland; 2 ARTORG Centre for Biomedical Engineering Research, Faculty of Medicine, University of Bern, Bern, Switzerland; 3 Endo-Team, Birshof Hospital, Muenchenstein, Switzerland; 4 Institute for Social and Preventive Medicine, University of Bern, Bern, Switzerland; 5 ChristenOrtho, Duedingen, Switzerland

**Keywords:** Knee, Arthroplasty, Unicompartmental, Periprosthetic joint infection, PJI, SIRIS, Joint registry, DAIR, Implant retention

## Abstract

**Introduction:**

Periprosthetic joint infection (PJI) remains a severe complication in arthroplasty. Unicompartmental knee arthroplasty (UKA) may have lower PJI rates than total knee arthroplasty (TKA) because of smaller implants and less extensive surgical exposure. However, PJI treatment after UKA is challenging due to restricted debridement and limited options for local antibiotic delivery. This study compared the revision rate for PJI and the failure rate of implant-retaining revision for PJI between UKA and TKA in the Swiss national joint registry (SIRIS).

**Methods:**

A retrospective analysis was conducted using SIRIS data from 2012 to 2024, examining the first revision after primary UKA or TKA and the re-revision rate after debridement with isolated inlay exchange for PJI. Both analyses assessed revisions for any cause and specifically for PJI. Kaplan-Meier survival curves and hazard ratios (HR) were calculated.

**Results:**

Among 35’286 primary UKA and 188’952 primary TKA, 149 and 1’546 were revised for PJI, respectively. Revision rates for any cause were higher for UKA than TKA (HR 1.29, *p* < 0.001), whereas PJI-related revisions were about half as frequent (HR 0.53, *p* < 0.001). Following implant-retaining revision for PJI, repeat revision rates increased more rapidly for UKA than TKA, reaching 34.8% and 32.1%, respectively (HR 1.56, *p* = 0.099). The statistical precision for UKA was limited by small numbers.

**Conclusions:**

In SIRIS, the revision rate for PJI after primary UKA was about half that after primary TKA, while the revision rate for any cause was higher. These findings support the hypothesis that smaller implants and less extensive surgery may be associated with lower infection risk. Despite limited debridement options, implant-retaining revision for PJI after UKA was as successful as after TKA. Nonetheless, failure rates for such procedures remain high in Switzerland, at roughly one-third.

**Supplementary Information:**

The online version contains supplementary material available at 10.1007/s00402-025-06156-5.

## Introduction

Orthopaedic and trauma surgery relies heavily on implants, either for fracture fixation or for joint reconstruction [1]. Orthopaedic device-associated infections (ODAI) remain a severe complication of such procedures [2–5]. Revision arthroplasty due to periprosthetic joint infection (PJI) is associated with increased morbidity and mortality and inferior functional outcome compared to aseptic revision [6–9]. Similarly, fracture-related infection (FRI) also is associated with considerably worse outcomes than uncomplicated internal fixation [5, 10, 11]. Moreover, systemic antibiotics required to treat ODAI or PJI carry a high risk of severe adverse events [12].

Microbial adherence and biofilm formation on implant surfaces are key elements in the development of ODAI [1, 13–15]. Intuitively, larger implants, having larger surface area, would be more susceptible to infection. Indeed, larger implants such as tumor- or megaprostheses, or complex internal fixation using more and larger implants, are associated with higher infection rates than arthroplasty using regular primary implants or smaller fracture-fixation devices [1, 16–20]. However, this may also reflect confounding risk factors, such as the severity of soft tissue trauma, extent of the surgical exposure, longer operative times in complex cases, preexisting scar tissue formation in case of revision, or any oncologic treatment like radio- or chemotherapy. Given limited data on ODAI risk relative to implant size, the data of the Swiss national arthroplasty registry (SIRIS) were explored to compare the revision risk for periprosthetic joint infection (PJI) of unicompartmental knee arthroplasty (UKA) versus total knee arthroplasty (TKA). The hypothesis was that UKA would be associated with a lower risk of revision for PJI than TKA. Respectively, the null-hypothesis for statistical analysis was that there would be no difference.

Furthermore, infection following UKA poses unique challenges compared to TKA, including limited debridement and local antibiotic delivery options, and a concomitant native joint septic arthritis of the other compartments within the knee [3, 21–23]. Consequently, the outcome of debridement, antibiotics and implant-retention (DAIR) procedures of UKA was compared to TKA within the same dataset as a secondary study question. The hypothesis was that the failure rate, as assessed by the rate of repeated revision, would be higher after DAIR procedures performed for PJI after UKA than for TKA. Respectively, the null-hypothesis for statistical analysis was that there would be no difference.

## Patients and methods

This study was a retrospective analysis of the data available in the Swiss national joint registry SIRIS from 1st January 2012 to 31st December 2024. Details regarding data collection, data management and coverage of the registry are available in the annual report [24]. The registry satisfies national data protection regulations and benefits of the necessary ethical committee approval. In addition, each patient included in the joint registry had to give their personal consent for anonymised data use.

Categorical data were described using counts and percentages, with comparison performed using the chi-squared test. Continuous data were compared with the unpaired t-test. Statistical significance was accepted for *p* < 0.05.

The case report forms used in SIRIS underwent modifications over time. For the analysis of the revision rates for any cause, all versions of the revision case report forms were considered. This provided a follow-up of up to 10 years for the revision rate for all causes. For the analysis of the distribution of specific reasons for revision, the analysis was limited to revision case report forms version 2015 and onwards. Therefore, only up to 8 years of follow-up were possible for this sub-analysis.

The cumulative revision risk over time was estimated using Kaplan-Meier (KM) survival curves and hazard ratios (HR) were calculated to compare outcomes of UKA versus TKA. All diagnoses were considered for the index arthroplasty, not only primary osteoarthritis. Both the revision risk for any cause as well as the risk of revision for PJI were examined, considering only the first revision after the index primary arthroplasty. Additionally, the cumulative revision risk over time for all reasons of repeated revision after DAIR procedures was studied. DAIR was defined as a revision due to infection with sole exchange of the inlay. Revision risks over time and risks of repeated revision over time after a DAIR procedure were assessed by comparing Kaplan-Meier estimates with 95% confidence intervals (CI) and Cox proportional hazard models risk-adjusted for age, sex, body mass index (BMI), and American Society of Anaesthesiology (ASA) class. Models for cumulative revision risk over time (first revisions) were also adjusted with an indicator for secondary osteoarthritis (using the SIRIS definition which groups OA after meniscus surgery with primary OA, secondary osteoarthritis including cases caused by inflammatory disease, post-infection, post-traumatic cases including after ligamentous injury, as well as cases secondary to patella instability) and deciles of total hospital and surgeon volume (2012–2024). Hospital volume was always included, surgeon volume only as an alternative for sensitivity testing. Due to much smaller sample sizes, only age, sex, BMI, and ASA were applied as potential confounders in re-revision models.

## Results

A total of 35’286 primary UKA and 188’952 primary TKA were available for analysis. Basic demographic and surgical data are provided in Table 1. Of these cases, 2’785 UKA (7.9%) and 9’274 TKA (4.9%) had been revised for any cause until 31.12.2024. Regarding PJI, the numbers were 149 UKA (0.4%) and 1’546 TKA (0.8%), considering all types of revisions, respectively treatment options. While the revision rate for any cause for UKA was higher than for TKA (HR 1.29, *p* < 0.001), the revision rate for PJI was only half as high (HR 0.53, *p* < 0.001). Other confounders had very little influence on the revision rate for any cause. Revision risk declined continuously with age (HR 0.97, *p* < 0.001) and is particularly elevated in patients with ASA 3+ (HR 1.46, *p* < 0.001). Also, larger hospitals and more experienced surgeons tend to have lower revision rates. Revision risk for PJI was much higher in men (HR 2.17, *p* < 0.001) and ASA 3 + patients (HR 1.78, *p* < 0.001). It also rose continuously with BMI (HR 1.03, *p* < 0.001) and was higher in secondary OA (HR 1.29, *p* = 0.002). Hospital and surgeon volume had no systematic impact on revision risk for PJI. In both models, the presence of the confounding variables only had a very marginal moderating effect on the UKA vs. TKA main effect under investigation.

**Table 1:** Basic demographic and surgical data of the UKA and the TKA cases from 01.01.2012 to 31.12.2024 registered in SIRIS

Parameter	UKA		TKA			Statistics
Sex	n	(%)	n	(%)		
Female	17,000	(48.2)	112,582	(59.6)		
Male	18,286	(51.8)	76,368	(40.4)		
Total	35,286	(100)	188,950	(100)		*p* < 0.001^**a**^
Diagnosis	n	(%)	n	(%)		
Primary OA	29,919	(84.8)	162,815	(86.2)		
Osteonecrosis	1842	(5.2)	3174	(1.7)		
Sec. OA of inflammatory origin	75	(0.2)	1795	(0.9)		
Sec. OA after fracture	250	(0.7)	3403	(1.8)		
Sec. OA after lesion of ligament	577	(1.6)	8774	(4.6)		
Sec. OA after infection	13	(0.0)	301	(0.2)		
Sec. OA after meniscus surgery*	1932	(5.5)	6362	(3.4)		
Sec. OA caused by pat. instability*	223	(0.6)	688	(0.4)		
Other	455	(1.3)	1640	(0.9)		
Total	35,286	(100)	188,952	(100)		*p* < 0.001^**a**^
Morbidity (ASA)**	n	(%)	n	(%)		
ASA 1	4261	(15.1)	11,913	(7.9)		
ASA 2	18,710	(66.5)	94,507	(62.9)		
ASA 3	5115	(18.2)	43,138	(28.7)		
ASA 4/5	68	(0.2)	580	(0.4)		
Total	28,154	(100)	150,138	(100)		*p* < 0.001^**a**^
Component fixation	n	(%)	n	(%)		
All cemented	28,960	(86.6)	140,854	(74.6)		
All uncemented	3857	(11.5)	13,369	(7.1)		
Hybrid	450	(1.3)	33,831	(17.9)		
Reverse hybrid	177	(0.5)	804	(0.4)		
Total	33,444	(100)	188,858	(100)		*p* < 0.001^**a**^
Patella component	n	(%)	n	(%)		
Yes	588	(1.8)	60,447	(32.0)		
No	32,698	(98.2)	128,352	(68.0)		
Status after patellectomy	15	(0.0)	59	(0.0)		
Total	33,301	(100)	188,858	(100)		*p* < 0.001^**a**^
Age at primary operation						
Years mean (SD)	64.7	(10.1)	69.5	(9.4)		
Years median (range)	64.0	(20–99)	70.0	(17–102)		
Data availability (n)	35,278		188,894			*p* < 0.001^**b**^
BMI at primary operation**						
kg/m^2^ mean (SD)	28.3	(4.8)	29.3		(5.5)	
kg/m^2^ median (range)	28.0	(13–85)	29.0		(11–104)	
Data availability (n)	25,865		140,475			*p* < 0.001^**b**^

* = available since 2021; ** = available since 2015.

^a^ = chi-squared test; ^b^ = unpaired t-test

SD = standard deviation. OA = osteoarthritis. BMI = body mass index

**Fig. 1:**
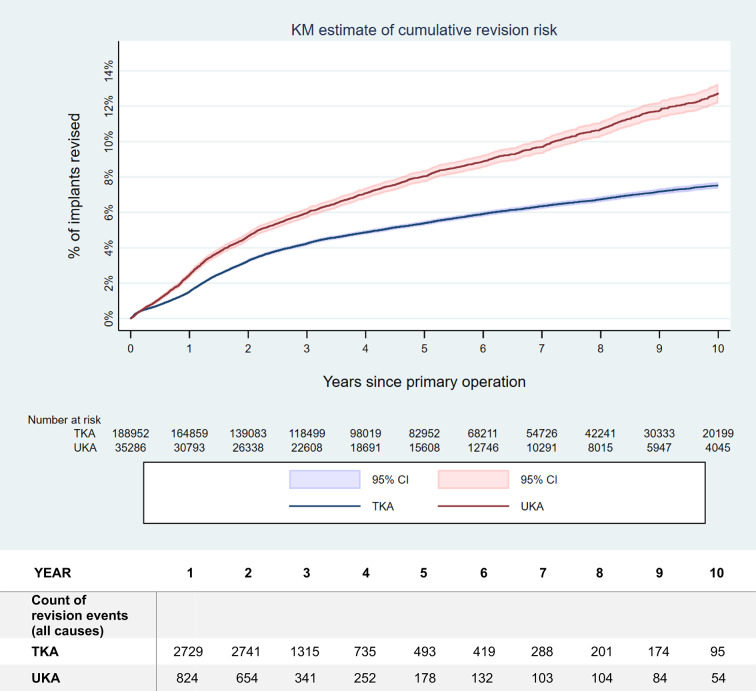
Kaplan-Meier (KM) estimation of the risk of revision over time for all causes after primary UKA, respectively TKA. The mean value as well as the 95% confidence interval (CI) are indicated. The revision rates for all causes differed significantly (hazard ratio (HR) 1.29, *p* < 0.001). Please note the numbers at risk, indicated below the figure

**Fig. 2:**
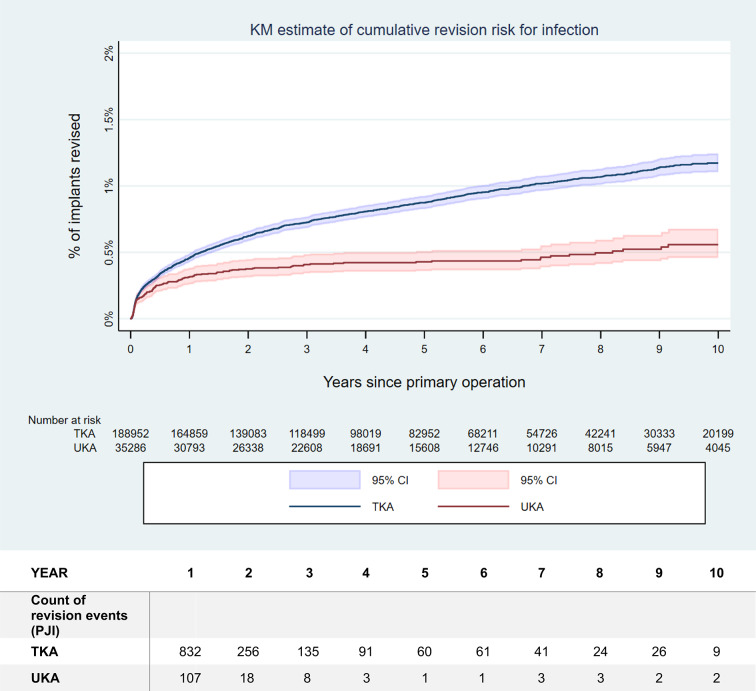
Kaplan-Meier (KM) estimation of the risk of revision over time for PJI after primary UKA, respectively TKA. The mean value as well as the 95% confidence interval (CI) are indicated. The revision rates for PJI differed significantly (hazard ratio (HR) 0.53, *p* < 0.001). Please note the numbers at risk, indicated below the figure

The risk of revision over time for all causes, as well as specifically for PJI, is illustrated in Fig. 1, respectively Fig. 2. Detailed numbers of the revision rates for any cause as well as for the specific reasons for revision over time are provided in Tables 2 and 3. Please note the difference in maximal duration of follow-up between the analysis of the revision rate for any causes and the analysis of the individual causes of revision. This was due to the latter being limited to details provided only on versions 2015 and later of the case report form.

**Table 2:** Risk of revision over time for all causes of revision after primary UKA and TKA, respectively for repeated revision after DAIR procedure

	1 year	2 years	3 years	4 years	5 years	6 years	7 years	8 years	9 years	10 years
*Revision risk (all causes)*										
UKA	2.5 (2.3–2.6)	4.7 (4.4–4.9)	6.0 (5.7–6.2)	7.1 (6.8–7.4)	8.0 (7.7–8.4)	8.9 (8.5–9.3)	9.7 (9.3–10.1)	10.7 (10.3–11.1)	11.8 (11.3–12.3)	12.7 (12.2–13.3)
TKA	1.5 (1.5–1.6)	3.3 (3.2–3.4)	4.2 (4.1–4.3)	4.9 (4.8-5.0)	5.4 (5.3–5.5)	5.9 (5.8-6.0)	6.4 (6.2–6.5)	6.7 (6.6–6.9)	7.2 (7.0-7.3)	7.5 (7.4–7.7)
*Re-Revision risk after DAIR (all causes)*										
UKA	32.1 (23.2–43.3)	34.8 (25.6–46.2)	34.8 (25.6–46.2)	34.8 (25.6–46.2)	34.8 (25.6–46.2)	34.8 (25.6–46.2)	34.8 (25.6–46.2)			
TKA	19.6 (17.2–22.2)	23.9 (21.3–26.9)	26.1 (23.3–29.2)	27.2 (24.2–30.3)	27.5 (24.5–30.7)	28.4 (25.3–31.8)	29.9 (26.5–33.6)	29.9 (26.5–33.6)	32.1 (27.7–37.0)	32.1 (27.7–37.0)

Mean values with 95% confidence intervals are provided. Up to 10 years of follow-up were available, as all revision case report form variants could be considered for this analysis. Revision rates are provided only if at least 10 cases at risk were available

**Table 3:** Risk for revision over time by reason for revision after primary UKA, respectively TKA, as registered in SIRIS up to 31.12.2024

Reason for revision/ follow-up	1 year	2 years	3 years	5 years	6 years	7 years	8 years
*UKA*							
Patella problems	0.1 (0.1–0.2)	0.4 (0.3–0.5)	0.5 (0.4–0.6)	0.7 (0.6–0.8)	0.7 (0.6–0.9)	0.8 (0.7-1.0)	0.9 (0.7-1.0)
Infection	0.3 (0.3–0.4)	0.4 (0.3–0.5)	0.4 (0.3–0.5)	0.4 (0.4–0.5)	0.5 (0.4–0.5)	0.5 (0.4–0.6)	0.5 (0.4–0.6)
Pain of unknown origin	0.1 (0.1–0.1)	0.2 (0.2–0.3)	0.3 (0.3–0.4)	0.4 (0.3–0.5)	0.5 (0.4–0.6)	0.5 (0.4–0.6)	0.6 (0.4–0.7)
Femorotibial instability	0.2 (0.1–0.2)	0.4 (0.3–0.5)	0.5 (0.4–0.6)	0.8 (0.7–0.9)	0.8 (0.7-1.0)	0.9 (0.8–1.1)	1.0 (0.9–1.2)
Loosening tibial component	0.7 (0.6–0.8)	1.5 (1.3–1.6)	1.9 (1.7–2.1)	2.5 (2.3–2.7)	2.8 (2.5-3.0)	3.0 (2.7–3.3)	3.3 (3.0-3.6)
Joint stiffness / arthrofibrosis	0.1 (0.0-0.1)	0.1 (0.1–0.2)	0.1 (0.1–0.2)	0.2 (0.1–0.3)	0.2 (0.1–0.3)	0.2 (0.1–0.3)	0.2 (0.1–0.3)
Malposition / wrong size	0.2 (0.1–0.2)	0.4 (0.3–0.4)	0.4 (0.3–0.5)	0.5 (0.4–0.6)	0.6 (0.5–0.7)	0.6 (0.5–0.8)	0.7 (0.6–0.8)
Other reasons	1.0 (0.9–1.1)	1.7 (1.6–1.9)	2.3 (2.1–2.5)	3.2 (3.0-3.5)	3.6 (3.4–3.9)	4.1 (3.8–4.4)	4.6 (4.2-5.0)
*TKA*							
Patella problems	0.4 (0.3–0.4)	1.2 (1.2–1.3)	1.7 (1.6–1.7)	2.2 (2.1–2.2)	2.4 (2.3–2.5)	2.6 (2.5–2.7)	2.8 (2.7–2.9)
Infection	0.5 (0.4–0.5)	0.6 (0.6–0.7)	0.7 (0.7–0.8)	0.9 (0.8–0.9)	1.0 (0.9-1.0)	1.0 (1.0-1.1)	1.1 (1.0-1.1)
Pain of unknown origin	0.0 (0.0–0.0)	0.1 (0.1–0.1)	0.1 (0.1–0.1)	0.1 (0.1–0.1)	0.1 (0.1–0.1)	0.1 (0.1–0.1)	0.1 (0.1–0.1)
Femorotibial instability	0.2 (0.2–0.3)	0.6 (0.6–0.7)	0.9 (0.8–0.9)	1.1 (1.1–1.2)	1.2 (1.2–1.3)	1.3 (1.2–1.4)	1.4 (1.3–1.5)
Loosening tibial component	0.1 (0.1–0.1)	0.3 (0.3–0.4)	0.5 (0.4–0.5)	0.7 (0.7–0.8)	0.8 (0.8–0.9)	0.9 (0.9-1.0)	1.0 (0.9–1.1)
Joint stiffness / arthrofibrosis	0.1 (0.1–0.2)	0.3 (0.3–0.3)	0.3 (0.3–0.4)	0.4 (0.4–0.5)	0.4 (0.4–0.5)	0.5 (0.4–0.5)	0.5 (0.4–0.5)
Malposition / wrong size	0.1 (0.1–0.1)	0.3 (0.2–0.3)	0.4 (0.3–0.4)	0.5 (0.4–0.5)	0.5 (0.5–0.6)	0.5 (0.5–0.6)	0.6 (0.5–0.6)
Other reasons	0.3 (0.3–0.3)	0.4 (0.4–0.5)	0.5 (0.5–0.6)	0.6 (0.6–0.7)	0.7 (0.6–0.7)	0.7 (0.7–0.8)	0.8 (0.7–0.8)

Only revisions recorded with the SIRIS case report form version 2015 or later were considered for this analysis to ensure comparability. Mean values with 95% confidence intervals are provided

Furthermore, 12 component-preserving revisions for PJI of primary UKA without component exchange and 69 of such TKA revisions could be identified. Adding these into the analysis did not change the conclusions. As not corresponding to formal DAIR procedures, these revisions were not described more in detail.

**Fig. 3:**
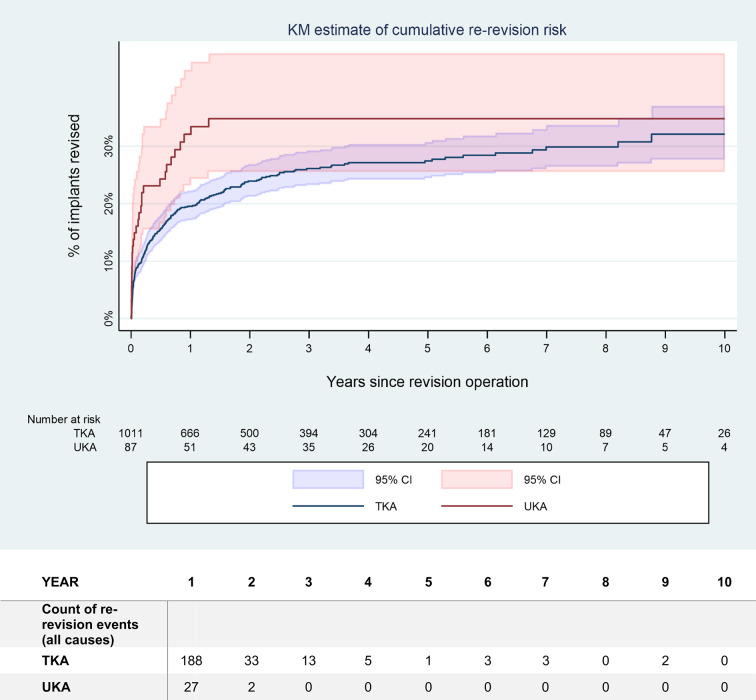
Kaplan-Meier (KM) estimation of the risk of repeated revision over time for all causes after DAIR procedure (with and without exchange of the liner) after primary UKA, respectively TKA. The mean value as well as the 95% confidence interval (CI) are indicated. There was no statistically significant difference between both groups (hazard ratio (HR) 1.56; *p* = 0.099). Please note the numbers at risk, indicated below the figure

**Fig. 4:**
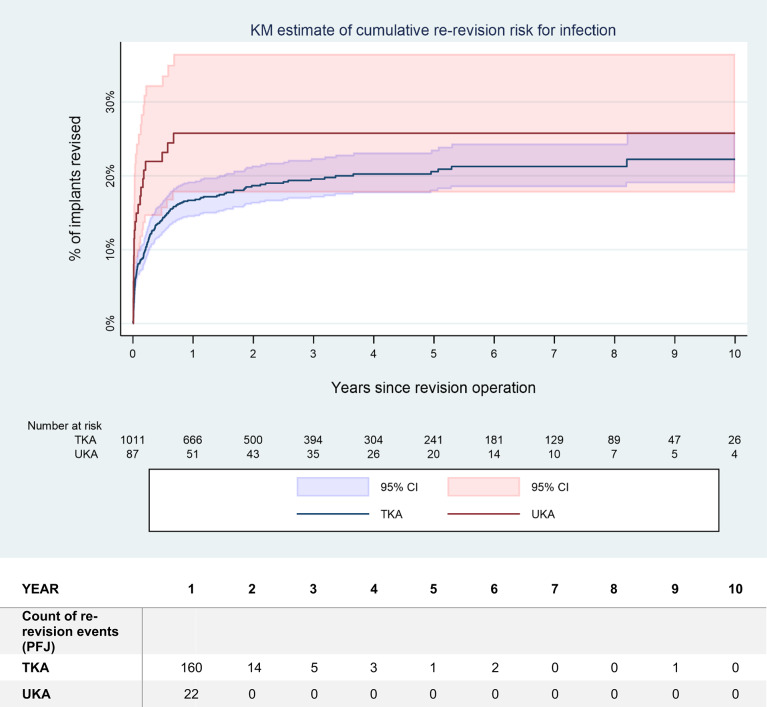
Estimated risk of repeated revision over time for PJI after DAIR procedure after primary UKA, respectively TKA. The mean value as well as the 95% confidence interval (CI) are indicated. There was no statistically significant difference between both groups (hazard ratio (HR) 1.49; *p* = 0.202). Please note the numbers at risk, indicated below the figure

Among the revision for PJI, 87 UKA (0.2% of total, 58.4% of revisions for PJI) and 1’011 TKA (0.5% of total, 65.4% of revisions for PJI) were treated by DAIR procedures. The rate of repeated revision following a DAIR procedure for PJI of the knee are indicated in Tables 3 and 4. The overall risk of repeated revision reached 34.8% (CI 25.6–46.2%) within 2 years for UKA, staying at this level thereafter, whereas the increase was slightly slower for TKA and continuing up to 9 years of follow-up, finally reaching 32.1% (CI 27.7–37.0%). There was no statistically significant difference between UKA and TKA, both for repeated revision for all causes (HR 1.56, *p* = 0.099) (Fig. 3) as for repeated revision for persistent or recurrent PJI (HR 1.496, *p* = 0.202) (Fig. 4). Reasons for repeated revision were mainly persistent PJI (Table 3). However, statistical precision of the UKA value was low due to small numbers, with *n* = 87 in the UKA group, whereas *n* = 1’011 were in the TKA group.

**Table 4:** Risk for repeated revision over time by reason for revision after DAIR procedure after primary UKA, respectively TKA, as registered in SIRIS up to 31.12.2024

Reason for re-revision/follow-up	1 year	2 years	3 years	5 years	6 years	7 years	8 years
*UKA*							
Patella problems	0.0 (0.0–0.0)	0.0 (0.0–0.0)	0.0 (0.0–0.0)	0.0 (0.0–0.0)			
Infection	26.0 (17.6–37.4)	26.0 (17.6–37.4)	26.0 (17.6–37.4)	26.0 (17.6–37.4)			
Pain of unknown origin	0.0 (0.0–0.0)	0.0 (0.0–0.0)	0.0 (0.0–0.0)	0.0 (0.0–0.0)			
Femorotibial instability	1.6 (0.2–11.1)	1.6 (0.2–11.1)	1.6 (0.2–11.1)	1.6 (0.2–11.1)			
Loosening tibial component	4.0 (1.0–15.0)	6.1 (2.0-17.9)	6.1 (2.0-17.9)	6.1 (2.0-17.9)			
Joint stiffness / arthrofibrosis	0.0 (0.0–0.0)	0.0 (0.0–0.0)	0.0 (0.0–0.0)	0.0 (0.0–0.0)			
Malposition / wrong size	0.0 (0.0–0.0)	0.0 (0.0–0.0)	0.0 (0.0–0.0)	0.0 ( (0.0–0.0)			
Other reasons	6.2 (2.0-18.1)	8.5 (3.3–21.2)	8.5 (3.3–21.2)	8.5 (3.3–21.2)			
*TKA*							
Patella problems	1.3 (0.7–2.4)	2.7 (1.7–4.3)	3.6 (2.3–5.5)	3.6 (2.3–5.5)	3.6 (2.3–5.5)	3.6 (2.3–5.5)	3.6 (2.3–5.5)
Infection	17.2 (14.8–19.8)	19.0 (16.5–21.8)	19.8 (17.3–22.7)	21.0 (18.2–24.1)	21.8 (18.9–25.1)	21.8 (18.9–25.1)	21.8 (18.9–25.1)
Pain of unknown origin	0.0 (0.0–0.0)	0.0 (0.0–0.0)	0.2 (0.0-1.7)	0.2 (0.0-1.7)	0.2 (0.0-1.7)	0.2 (0.0-1.7)	0.2 (0.0-1.7)
Femorotibial instability	0.8 (0.3–1.7)	1.7 (0.9-3.0)	2.2 (1.2–3.8)	2.2 (1.2–3.8)	2.2 (1.2–3.8)	3.8 (1.9–7.5)	3.8 (1.9–7.5)
Loosening tibial component	0.7 (0.3–1.6)	1.1 (0.5–2.2)	1.6 (0.8–3.1)	1.6 (0.8–3.1)	1.6 (0.8–3.1)	3.2 (1.5-7.0)	3.2 (1.5-7.0)
Joint stiffness / arthrofibrosis	0.9 (0.4–1.9)	1.4 (0.7–2.7)	1.4 (0.7–2.7)	1.4 (0.7–2.7)	1.4 (0.7–2.7)	1.4 (0.7–2.7)	1.4 (0.7–2.7)
Malposition / wrong size	0.6 (0.2–1.6)	1.0 (0.4–2.2)	1.0 (0.4–2.2)	1.0 (0.4–2.2)	1.0 (0.4–2.2)	1.0 (0.4–2.2)	1.0 (0.4–2.2)
Other reasons	1.3 (0.7–2.3)	1.4 (0.8–2.6)	1.7 (0.9-3.0)	2.0 (1.1–3.6)	2.0 (1.1–3.6)	2.0 (1.1–3.6)	2.0 (1.1–3.6)

Only revisions recorded with the SIRIS case report form version 2015 or later were considered for this analysis to ensure comparability. Mean values with 95% confidence intervals are provided. Data are provided only if at least 10 cases at risk were available

Considering small numbers within the various subgroups and therefore limited contribution, results from the multivariate Cox models are available as supplementary material. While there may have been a selection bias favouring UKA, with younger and fitter patients than in the TKA group, the most important influence on the risk for revision for all causes, respectively for revision for PJI, was the type of arthroplasty. However, this analysis must be considered with caution due to statistical limitations.

## Discussion

This study provides valuable insights into the occurrence and treatment of PJI after UKA, especially given the paucity of data available on this topic [3, 21]. Our findings show that UKA was associated with a significantly smaller risk of revision for PJI than TKA (HR 0.53, *p* < 0.001). Although the study is observational, considering the similarities among the patients in both groups (Table 1) and regarding local tissue conditions, this may indeed be considered as an indicator for smaller implants being associated with a lower risk of infection. Since UKA are also associated with better functional outcomes than TKA [25–28], these findings represent an additional argument for preferring UKA whenever anatomically feasible, despite associated technical challenges and higher early revision rates [29, 30]. There was however only limited possibility to adjust for confounding factors, the analysis being limited to the data registered routinely in SIRIS. While it may be accepted that younger and fitter patients were preferentially chosen for UKA, showing with a mean age difference of 5 years and a mean BMI difference of 1 kg/m^2^, small counts in certain subgroups limited the statistical precision of the multivariate analysis. Therefore, results of the Cox proportional hazard models have been provided as supplementary material. Furthermore, younger age is a recognized risk factor for PJI after TKA - albeit a small one after controlling for ASA score and on the boundary of statistical significance – which would be contrary to the observation in this study of lower PJI risk in the UKA group [31].

Despite the lower revision risk for PJI (HR 0.53, *p* < 0.001), the revision risk for any cause after UKA was higher than after TKA (HR 1.29, *p* < 0.001). This aligns with previous observations, partly explained by technical factors, residual symptoms from degenerative disease in the untreated compartments, and the lower threshold for conversion of UKA to TKA compared to TKA revision [29, 30, 32]. Nevertheless, the revision rates after UKA observed in SIRIS are consistent with other reports, reinforcing our observations [29, 30, 33, 34]. While the difference in PJI risk had already been identified in previous reports with larger sample size, this had not been highlighted so far, possibly as overshadowed by the higher revision rate for all causes associated with UKA.

Although infection following UKA are less common than after TKA, they present unique challenges. Infection combines PJI with a native joint septic arthritis [22]. The typical paramedian short quadriceps-sparing approach for UKA restricts or prevents access to the posterior aspect or to the other compartments of the knee joint. Any posterior exposure may even be impossible in UKA models with a monobloc tibial component [23]. Local application of antiseptic solutions or antibiotics is restricted, as the cartilage in the native compartments limits drug delivery due to toxicity issues [35–41] and as the carrier materials may cause mechanical damage, even calcium sulphate being hard enough to cause some wear on metallic implants [42–44]. Postinflammatory destruction of the cartilage may also lead to disease progression in the native compartments [45], necessitating conversion to TKA even after successful infection management [22]. Due to these limitations, the 2018 International Consensus Meeting in Philadelphia 2018 even recommended against attempting DAIR in case of PJI after UKA, favouring conversion procedures instead [3, 21, 22]. Considering the paucity of data available at the time of this consensus regarding DAIR in UKA, newer results may well contradict this expert opinion. In this study, the rate of repeated revision after DAIR procedure for PJI following primary UKA reached 34.8% (25.6–46.2%) at 3 years, predominantly due to infection (Table 4). This may seem elevated but was not significantly higher (HR 1.56; *p* = 0.099) than for TKA with 26.1% (23.3–29.2%) (Table 4; Figs. 3 and 4).

While discussions about PJI often emphasize failure rates, the success rates are also meaningful. A failure rate of 30% after DAIR may seem high, but on the other hand, it indicates that more than two thirds of the cases were successfully managed. In case of successful DAIR, functional results comparable to primary arthroplasty without infection are achievable [46–48]. Therefore, the authors confirm that DAIR can be a reasonable treatment option for PJI after UKA [49–52] and should not lead automatically to revision with conversion to TKA, contrary to current recommendations [3, 21, 22]. However, all reasonable efforts should be put into early detection of infection, considering the importance of time lapse in the success rates of DAIR procedures [23, 33, 53–55]. The duration of symptoms before revision could not be evaluated in this study, as such data were not registered in SIRIS. Beside the unclear time lapse between first symptoms of PJI and revision, SIRIS also does not provide any information about the causative microorganism, which could also influence results of DAIR and recurrent or persistent of PJI [23, 56, 57]. While the rate of 26.1% (23.3–29.2%) of repeated revision at 3 years after DAIR for PJI after TKA may appear high, it remains below or similar to the failure rates reported in larger or multicentric studies [21, 33, 56, 58, 59]. Individual, monocentric studies may report higher success rates. However, there is a well-known positive publication bias in medicine, leading to inflation of results in smaller studies compared to national registries [60–62].

Arthroplasty registries, including SIRIS, face well-known limitations regarding the accuracy of reporting the reason for revision [3, 63]. A diagnosis of PJI, for example, is often recognized only postoperatively due to delays in tissue samples and retrievals analyses [64–69]. Because case report forms are typically completed shortly after surgery, this delay can lead to misclassification, often as aseptic loosening or pain of unknown origin. Correct classification of the underlying cause for revision after definitive microbiological and histological results are available may be uncertain. Although SIRIS permits registering multiple diagnoses at revision, only the preponderant cause of revision may have been recognized and documented. For this reason, both the risk of revision for any cause as well as the specific risk of revision for PJI have been examined, following guidelines by the benchmarking working group of the International Society of Arthroplasty Registries [63]. On the other hand, SIRIS has excellent coverage (98.5%), greatly enhancing the reliability of the results [24]. Moreover, the analyses of both the revision rate for any cause as well as specifically for PJI, align closely. Furthermore, the classification of revisions in SIRIS may be confusing, as DAIR procedures must be identified as component-preserving revision with additional exchange of the inlay. As mentioned, there would have been 81 more component-preserving revisions for infection registered, but without any component exchange. Including these cases did not alter the conclusions. As not formal DAIR procedures, they have been left out of this report. Overall, no major bias is to be expected. Small numbers did not allow to consider the various subtypes of UKA (medial, lateral, patella-femoral joint) separately.

No particular methods were applied to correct for missing data. Thus, a missing completely at random (MCAR) scenario was assumed. The pattern of missing BMI and ASA values in SIRIS is characterized by a strong time component. When both variables were introduced in 2015, approx. 30% of BMI and 12% of ASA values were missing, but those rates fell gradually to under 4% by 2024, as more hospitals started to provide ASA and BMI. The impact of case-wise-deletion in modelling SIRIS data is therefore one of reducing the relative weight of older cases in the analysis. In other words, if results should be biased, they are biased towards more recent clinical results, which we deemed unproblematic for analyses of PJI.

## Conclusion

While the study is observational and therefore limited to documentation of associations, the observations are consistent with the hypothesis that smaller implants may be associated with lower rates of ODAI/PJI. This may provide a valuable reason to favour UKA over TKA, whenever possible, particularly as UKA are associate with less local and general morbidity, a lower mortality and may have better functional results. This study however confirms that the revision rate for all causes is higher after UKA than after TKA. Despite surgical limitations in implant-retaining UKA revision, the rate of repeated revision after DAIR was not significantly different for UKA and TKA, but was around one third in both groups.

## Supplementary Information

Below is the link to the electronic supplementary material.

Supplementary Material 1

## Data Availability

The data that support the findings of this study are available from the authors upon reasonable request but restrictions apply to the availability of these data, which were used under license from the Swiss Arthroplasty Registry SIRIS for the current study, and so are not publicly available.
