# Supplementary material for: Data from the Swiss National Arthroplasty Registry SIRIS suggest that unicompartmental knee arthroplasty is associated with a lower risk of periprosthetic joint infection than total knee arthroplasty
Source: Arch Orthop Trauma Surg. 2026 Mar 4;146(1):95. doi: 10.1007/s00402-025-06156-5 (PMC12960357; doi:10.1007/s00402-025-06156-5)
Supplement: Supplementary file 1 — Supplementary Material 1 [file 402_2025_6156_MOESM1_ESM.docx]

**Cox model 1: TKA vs UKA - all first revisions**

|  | Coefficient | p-value | 95% lower bound | 95% upper bound |
| --- | --- | --- | --- | --- |
| TKA (reference) |  |  |  |  |
| UKA | 1.294283 | 0 | 1.222348 | 1.370451 |
| Age at primary operation | 0.966776 | 0 | 0.9643925 | 0.9691654 |
| BMI score | 0.9981235 | 0.397 | 0.9937962 | 1.00247 |
| Female (reference) |  |  |  |  |
| Male | 0.9626878 | 0.102 | 0.9197358 | 1.007646 |
| ASA1, no disturbance (reference) |  |  |  |  |
| ASA2, mild/moderate | 1.15969 | 0 | 1.070016 | 1.25688 |
| ASA3+, severe | 1.463411 | 0 | 1.335724 | 1.603304 |
| Primary OA (reference) |  |  |  |  |
| Secondary OA | 1.105368 | 0.003 | 1.034062 | 1.181591 |
| Hospital volume percentile 1 – smallest (reference) |  |  |  |  |
| 2 | 0.7031161 | 0 | 0.6327741 | 0.7812776 |
| 3 | 0.9744431 | 0.6 | 0.8846592 | 1.073339 |
| 4 | 0.7840553 | 0 | 0.7089859 | 0.8670733 |
| 5 | 0.9281639 | 0.123 | 0.8442353 | 1.020436 |
| 6 | 0.8236562 | 0 | 0.7482026 | 0.9067191 |
| 7 | 0.903996 | 0.045 | 0.819 | 0.997813 |
| 8 | 0.8841183 | 0.011 | 0.8040424 | 0.9721692 |
| 9 | 0.8430964 | 0.001 | 0.7595217 | 0.9358672 |
| 10 | 0.7232731 | 0 | 0.6529861 | 0.8011258 |

**Cox model 2: TKA vs UKA - first revision for PJI**

|  | Coefficient | p-value | 95% lower bound | 95% upper bound |
| --- | --- | --- | --- | --- |
| TKA (reference) |  |  |  |  |
| UKA | 0.533677 | 0 | 0.435061 | 0.6546464 |
| Age at primary operation | 0.9931987 | 0.046 | 0.9865646 | 0.9998773 |
| BMI score | 1.029695 | 0 | 1.018749 | 1.040759 |
| Female (reference) |  |  |  |  |
| Male | 2.168737 | 0 | 1.92521 | 2.443069 |
| ASA1, no disturbance (reference) |  |  |  |  |
| ASA2, mild/moderate | 1.061647 | 0.624 | 0.8357947 | 1.348531 |
| ASA3+, severe | 1.78413 | 0 | 1.3833 | 2.301106 |
| Primary OA (reference) |  |  |  |  |
| Secondary OA | 1.292674 | 0.002 | 1.095334 | 1.525568 |
| Hospital volume percentile 1 – smallest (reference) |  |  |  |  |
| 2 | 0.6795792 | 0.004 | 0.5208251 | 0.8867235 |
| 3 | 0.9111407 | 0.462 | 0.7109955 | 1.167627 |
| 4 | 0.8560664 | 0.219 | 0.6680608 | 1.096981 |
| 5 | 0.9621734 | 0.751 | 0.7584296 | 1.220651 |
| 6 | 0.6833586 | 0.003 | 0.5295336 | 0.8818684 |
| 7 | 1.053734 | 0.671 | 0.8273296 | 1.342095 |
| 8 | 0.7555425 | 0.034 | 0.5834072 | 0.9784667 |
| 9 | 0.9187977 | 0.531 | 0.704979 | 1.197467 |
| 10 | 0.6901987 | 0.006 | 0.5311364 | 0.8968963 |

**Cox model 3: TKA vs UKA - all first re-revisions after DAIR**

|  | Coefficient | p-value | 95% lower bound | 95% upper bound |
| --- | --- | --- | --- | --- |
| TKA (reference) |  |  |  |  |
| UKA | 1.5551316 | 0.099 | 0.9200486 | 2.628594 |
| Age at revision operation | 0.9838381 | 0.060 | 0.9673038 | 1.0006551 |
| BMI score | 0.9937899 | 0.615 | 0.9699566 | 1.0182088 |
| Female (reference) |  |  |  |  |
| Male | 1.357439 | 0.074 | 0.9707816 | 1.8981 |
| ASA1, no disturbance (reference) |  |  |  |  |
| ASA2, mild/moderate | 0.7119826 | 0.520 | 0.2527078 | 2.0059495 |
| ASA3+, severe | 1.1979834 | 0.733 | 0.4240503 | 3.3844202 |

**Cox model 4: TKA vs UKA - all first re-revisions for PJI after DAIR**

|  | Coefficient | p-value | 95% lower bound | 95% upper bound |
| --- | --- | --- | --- | --- |
| TKA (reference) |  |  |  |  |
| UKA | 1.4927892 | 0.202 | 0.806723 | 2.7623105 |
| Age at revision operation | 0.9872105 | 0.190 | 0.9683881 | 1.0063988 |
| BMI score | 0.9988685 | 0.934 | 0.9726103 | 1.0258356 |
| Female (reference) |  |  |  |  |
| Male | 1.3141378 | 0.156 | 0.90062 | 1.9175215 |
| ASA1, no disturbance (reference) |  |  |  |  |
| ASA2, mild/moderate | 1.0574747 | 0.939 | 0.2515018 | 4.4463 |
| ASA3+, severe | 1.6829342 | 0.478 | 0.3993277 | 7.0925905 |
